# Time Slot Utilization for Efficient Multi-Channel MAC Protocol in VANETs

**DOI:** 10.3390/s18093028

**Published:** 2018-09-10

**Authors:** VanDung Nguyen, Tran Anh Khoa, Thant Zin Oo, Nguyen H. Tran, Choong Seon Hong, Eui-Nam Huh

**Affiliations:** 1 Department of Computer Science and Engineering, Kyung Hee University, Yongin-si, Gyeonggi-do 17104, Korea; ngvandung85@khu.ac.kr (V.D.N.); tzoo@khu.ac.kr (T.Z.O.); nguyen.tran@sydney.edu.au (N.H.T.); 2 Department of Electronics and Telecommunication Engineering, Faculty of Electrical and Electronics Engineering Ton Duc Thang University, Ho Chi Minh City 756636, Vietnam; trananhkhoa@tdtu.edu.vn; 3 School of Information Technologies, The University of Sydney, Sydney, NSW 2006, Australia

**Keywords:** VANET, multi-channel MAC, saturation throughput

## Abstract

In vehicular ad hoc networks (VANETs), many schemes for a multi-channel media access control (MAC) protocol have been proposed to adapt to dynamically changing vehicle traffic conditions and deliver both safety and non-safety packets. One such scheme is to employ both time-division multiple access (TDMA) and carrier-sense multiple access (CSMA) schemes (called a hybrid TDMA/CSMA scheme) in the control channel (CCH) interval. The scheme can adjust the length of the TDMA period depending on traffic conditions. In this paper, we propose a modified packet transmitted in the TDMA period to reduce transmission overhead under a hybrid TDMA/CSMA multi-channel MAC protocol. Simulation results show that a MAC protocol with a modified packet supports an efficient packet delivery ratio of control packets in the CCH. In addition, we analyze the hybrid TDMA/CSMA multi-channel MAC protocol with the modified packet under saturated throughput conditions on the service channels (SCHs). The analysis results show that the number of neighbors has little effect on the establishment of the number of time slots in TDMA periods and on SCHs under saturated throughput conditions.

## 1. Introduction

According to the World Health Organization, 100 million people die in traffic accidents worldwide annually, accounting for economic losses of $500 billion [1]. Therefore, safe transportation has become one of the most important global issues. Recently, intelligent transportation systems (ITSs) have been used to enable significant improvements in performance, traffic flow, and the efficiency of passenger and goods transportation [2]. Moreover, an ITS ensures more comfortable travel for passengers by providing infotainment along the road. The ITS targets utilization of ubiquitous sensing and wireless networking capabilities for intelligent management of the transportation system [3]. The vehicular ad hoc network (VANET) is one important segment of an ITS, which furnishes the quality and effectiveness of safety messages in future transportation systems. A VANET consists of a set of special vehicles and roadside units (RSUs). VANETs employ dedicated short-range communications (DSRC) for vehicle-to-vehicle (V2V) and vehicle-to-RSU (V2R) communications. V2V communications-based applications broadcast within a one-hop neighborhood. For instance, each vehicle periodically broadcasts information about its position, speed, heading, acceleration, turn signal status, and so on, to all vehicles within its one-hop neighborhood [4] in order to announce precrash sensing, blind spot warnings, emergency electronic brake lights, and cooperative forward collision avoidance. Similarly, an RSU periodically broadcasts V2R communications–based applications, such as curve speed warnings and traffic signal violation warnings, to all approaching vehicles, plus information related to traffic signal status and timing, road surface types, weather conditions, and so on [5]. The main properties of VANETs are variable network density, large-scale networks, a predictable mobility model, and rapid topology changes. Therefore, compared with other networks, VANETs have high rates of topology change, restrictions on vehicle movements due to road structures, and availability of ample energy sources and processing power. On the other hand, in wireless sensor networks (WSNs), energy consumption is a very important issue, and is considered a vital mechanism in most protocols.

In Wireless Access in Vehicular Environments (WAVE), the DSRC spectrum is divided into seven 10 MHz channels: one control channel (CCH) and six service channels (SCHs). The CCH is used for exchanging high-priority safety applications and network management, whereas SCHs mainly support non-safety information and entertainment applications. VANET applications have different quality of service (QoS) requirements, such as transmission delay and bandwidth. First, safety-related applications are related to the safety of people on the road, such as emergency braking, blind spot warnings, and precrash sensing. Hence, safety-related applications require reliable and fast broadcasting mechanisms, and each vehicle must periodically broadcast information like location, speed, and acceleration [5,6]. One such application is the beacon packet containing the vehicles location, speed, and acceleration [5,6], which is broadcast periodically by each vehicle. Second, traffic management services consist of intersection management, delay warnings, road congestion prevention, toll collection, and cooperative adaptive cruise control. Third, user-oriented services provide information, advertisements, and entertainment for passengers while traveling. User-oriented services have two basic applications: Internet connectivity and peer-to-peer applications [7,8]. However, safety services require both fast access and low delay, whereas user-oriented services require large bandwidth [9]. We briefly summarize the requirements for different applications in Table 1.

Media access control (MAC) plays an important role in supporting efficient broadcast services and in satisfying requirements for VANET applications. Many MAC protocols have been proposed to provide high-throughput systems for service applications and to guarantee strict transmission delays for safety applications. There are three main schemes, depending on the channel access method used: contention-based media access, such as IEEE 802.11p [12]; contention-free media access, such as TDMA-based MAC protocols; and hybrids of the two methods. First, contention-based MAC protocols allow the vehicles to access the channel randomly when they have data to transmit. However, the collision probability is high when the network load is high. In addition, they cannot guarantee QoS requirements for critical road safety applications. Unlike contention-based MAC protocols, contention-free MAC protocols allow each vehicle to access a channel by following a schedule of time slot frequency bands or code sequences [9]. To do so, they require a strict synchronized scheme between vehicles. In the same two-hop neighbor set that contention-free MAC protocols can support, the packets are transmitted without collisions. The major issues of contention-free and contention-based MAC protocols are as follows:

1. Contention-free protocols require a global positioning system (GPS) to support location and time information, which is used to synchronize the communicating vehicles. In addition, the high mobility of vehicles can affect the performance of these protocols.
2. Contention-free protocols cannot satisfy QoS requirements for real-time applications. When the vehicle density is high, these protocols provide poor performance.

To enhance QoS requirements and reduce the number of packet collisions, hybrid MAC protocols were proposed to try to combine these two mechanisms into a single architecture. Such an architecture includes two periods on the access channel: a random access period and a contention-free access period. The contention-free access period is used to transmit safety packets to all nodes without collisions by using TDMA-based access schemes. The contention-free access period is used to exchange WAVE service announcements, acknowledgments, and responses to service (WSA/ACK/RES) and piggyback service information and the identities of SCHs to be used. Moreover, nodes create a channel access schedule by broadcasting HELLO packets in the contention-free access period.

The performance of single-channel MAC protocols, such as IEEE 802.11p [12], degrades quickly with an increase in vehicle density. This is because of high contention and collisions due to the increase in the number of vehicles, and, hence, the number of transmissions. Thus, multi-channel MAC protocols based on IEEE 802.11p and IEEE 1609.4 standards have higher performance than that of single-channel MAC protocols in every key performance indicator [13]. Furthermore, the multi-channel MAC protocol supports not only reliable transmission packets with low latency but also provides maximum throughput for non-safety applications. Many multi-channel MAC protocols have been proposed for efficiency and reliability [14,15,16]. IEEE 1609.4 [14] is considered a default multi-channel MAC standard in the family of IEEE 1609 standards for VANETs. In [14], the standard was developed to efficiently coordinate channel access on the CCH and SCHs, called a globally synchronized channel coordination scheme, based on coordinated universal time (UTC). The channel time is divided into synchronization intervals with a fixed length of 100 ms. It consists of a CCH interval (CCHI) and an SCH interval (SCHI) each with a length of 50 ms. This scheme allows safety and non-safety application packets to be transmitted on different channels without missing important packets on the CCH. However, IEEE 1609.4 cannot utilize all SCH resources during the CCH interval.

This paper focuses on the multichannel hybrid media access control schemes, which are based on draft IEEE 802.11p and IEEE 1609.4 standards. Our contributions are as follows.

- We investigate the existing hybrid MAC protocols and discuss their benefits and limitations.
- We propose a modified announcement packet to reduce payload size of a packet transmitted in the TDMA period.
- We use a Markov chain and a stochastic process to establish the number of initial time slots in both the TDMA period and on the SCHs under the condition of saturated traffic load.
- We analyze the trade-off between time slot selection in both the TDMA period and on the SCHs under a saturated traffic load condition.
- We optimize time slot selection on the SCHs and on the CCH under a saturated throughput condition.
- The analysis results show that the number of neighbors has little effect on the establishment of the number of time slots in both TDMA periods and SCHs under a saturated throughput condition.

The rest of this paper is organized as follows. Section 2 gives a short survey of hybrid MAC protocols in VANETs. Section 3 describes the modified announcement packet in detail. Section 4 discusses a theoretical analysis of establishing the number of time slots in both the TDMA period and on the SCHs under a condition of saturated traffic load. The performance evaluation is presented in Section 5. Section 6 gives conclusions to this paper.

## 2. Related Works

**Figure 1:**
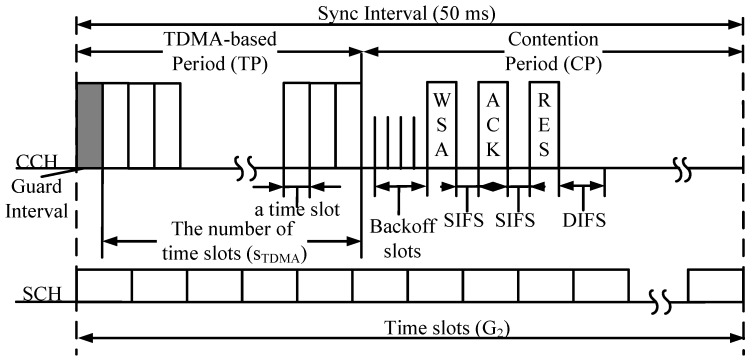
The considered multi-channel MAC protocol.

The multi-channel MAC protocol under consideration consists of TDMA periods and CSMA periods (called contention periods in this paper), as shown in Figure 1. In the TDMA period, each node has to broadcast its information, including safety applications, in its time slot. In the CSMA period, a node that has a non-safety packet will attempt to exchange WSA/ACK/RES messages and piggyback service information and the identities of SCHs to be used. There are two main schemes to reserve time slots in the TDMA period: the self-organization scheme, and broadcasting a HELLO packet in the CSMA period. Nevertheless, a node that wants to occupy a time slot has to know its two-hop neighbor information by receiving packets about their reserved time slots. This is because a node will obtain full information about its two-hop neighbors, and it can then choose an available time slot without any access collision (Access collision is defined as the collision happening when more than two nodes occupy the same time slot in the same two-hop neighborhood [4].). Therefore, a new node will broadcast its packet in its chosen time slot under the self-organization scheme. Otherwise, it broadcasts its HELLO packet during the contention-based period.

**Figure 2:**
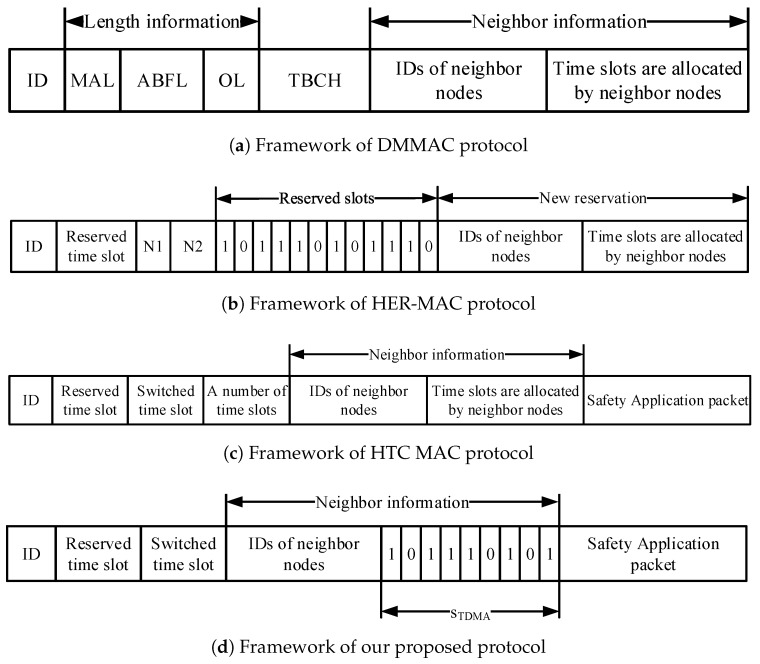
Comparison of frameworks used in hybrid MAC protocols.

Therefore, to provide full two-hop information on neighborhood vehicles for a target node, hybrid MAC protocols have been proposed in various frameworks to broadcast in the TDMA period of each time slot, as shown in Figure 2. Figure 2a shows fields included in a packet transmitted in the TDMA period under the dedicated multi-channel MAC (DMMAC) protocol [15]. This consists of length information (the maximum active length (MAL) of vehicles in its one-hop area, adaptive broadcast frame length (ABFL), and the maximum ABFL within a one-hop area (OL)), TBCH (which is used when the vehicle makes its active length as short as possible), and neighbor information. To reduce the overhead of a framework using the DMMAC protocol, the hybrid efficient and reliable MAC (HER MAC) protocol [16] uses a bitmap to represent the neighbor information, as shown in Figure 2b. N1 and N2 are the last time slots occupied by the one-hop neighbor nodes and by all neighbor nodes, respectively. The information helps a new node know the bitmap length of its one- and two-hop neighbors. Then, the new node can broadcast a HELLO packet including its ID and a reserved time slot to its one-hop neighborhood. Moreover, this information also helps a vehicle to shorten the TDMA period by eliminating the empty time slots [16]. However, there are many types of packets transmitted in the HER-MAC protocol. Hence, collision probability increases with an increase in the number of vehicles. The hybrid TDMA/CSMA MAC (HTC-MAC) protocol [17] was proposed to remove HELLO and SWITCH packets during the CSMA period. As shown in Figure 2c, a new field is added to the HTC-MAC framework to shorten the length of the TDMA period. Furthermore, HTC-MAC provides efficient time-slot acquisition by letting a new vehicle randomly choose an available time slot to broadcast the announcement (ANC) packet.

Time slot selection is an important issue for TDMA-based MAC protocols in VANETs. One of the well-known problems with TDMA-based MAC protocols, such as HER-MAC [16] and HTC-MAC [17], is transmission overhead when node density is high. To solve the transmission overhead problem, we propose a modified announcement packet to reduce the payload size of a packet transmitted in the TDMA period.

The saturation throughput of SCHs (or saturation of traffic load) is when all time slots on the SCHs are used after nodes successfully exchange WSA/ACK/RES in the CSMA period on the CCH. Time slots on the SCHs are used to transmit/receive large bandwidth–consuming applications, such as video downloads and map updates. Hence, the length of the TDMA period needs to ensure that all nodes can use sufficient bandwidth resources on the SCHs. This paper considers a way to optimize time slot selection on SCHs and on the CCH under conditions of saturation throughput and packet delay.

Table 2 summarizes a comparison between our proposal and the existing MAC protocols. Our proposal allows the adjustment of both TDMA and contention periods, based on vehicle density and data traffic conditions. By reducing the payload size of a packet transmitted in the TDMA period, our proposal can decrease the length of the TDMA period when vehicle density is high.

## 3. EMMAC: Efficient Multi-Channel MAC Protocol in VANETs

Each node in VANET under consideration has one transceiver which can switch between CCH and SCHs. A node tunes to CCH to transmit two kinds of information: (1) high-priority short application (such as periodic or event driven safety messages), and beacon packet which includes the vehicle’s position, speed, and acceleration [5] during the TDMA period; and (2) control information required for the nodes to determine which time slots they should access in SCHs in the CSMA period. In this paper, we present the modified announcement packet to reduce payload size of a packet transmitted in the TDMA period. Based on the modified announcement packets, we design an efficient multi-channel MAC protocol.

Nodes based on two-hop neighbors information adjust the length of the TDMA period. Two-hop neighbors information is collected by receiving the modified announcement packet (MANC) transmitted in the TDMA period including the time slots information. As shown in Figure 2d, MANC packet contains six fields: (i) node ID; (ii) its reserved time slot; (iii) a switched time slot; (iv) IDs of one-hop neighbor nodes; (v) bits representing the status of time slots in the TDMA period; and (vi) safety application packet. In the bits representing the status of time slots in the TDMA period, bit 0 means free time slot status; otherwise, bit 1 represents busy time slot status. Note that the number of bits is the length of time slots in the TDMA period.

### 3.1. TDMA Period Adjustment Scheme

To reduce the length of the TDMA period, under the HER-MAC [16] protocol, a switched node attempts to broadcast a SWITCH packet in the CSMA period to change its time slot. Under the DMMAC [15] protocol, each node, based on the length of the TDMA period and the last time slot that was occupied, reduces the length of the TDMA period in the next frame. Our proposed contention-free length-adjustment scheme under the efficient multi-channel MAC (EMMAC) protocol operates as follows:

1. Each node successfully receives MANC packets transmitted by one-hop neighbor nodes in the previous frame. Based on these MANC packets, each node has the status of all time slots and the number of time slots in the TDMA period.
2. Each node considers whether it should move to an available time slot without collisions to reduce the number of time slots in the TDMA period. If a node wants to change its time slot, it will randomly choose an available time slot. Then, this node will broadcast a switched time slot included in the MANC packet in its reserved time slots.
3. After one period of the sync-interval (100 ms under IEEE 1609.4 [14]), a switched node checks the MANC packets broadcast by one-hop neighbor nodes. If all neighbor nodes broadcast MANC packets including the updated information, it successfully acquires the new time slot, reducing the length of the TDMA period in the next frame.

**Figure 3:**
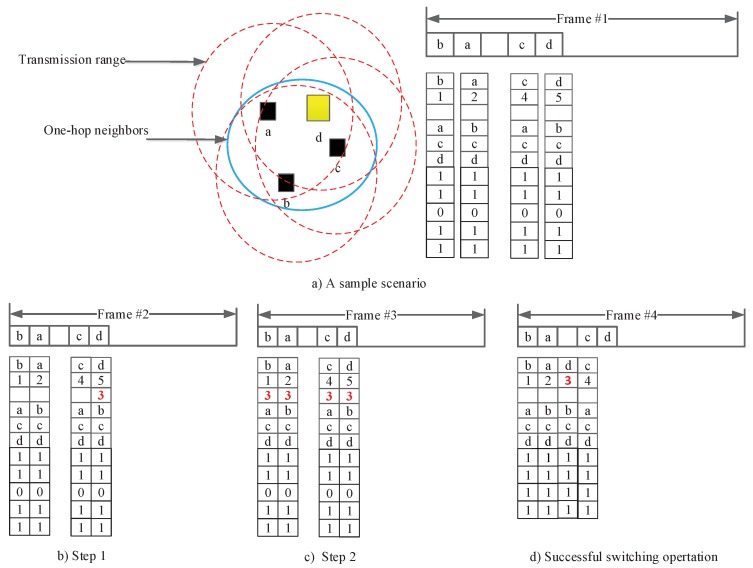
Operation of the adjustment scheme for node d. (**a**) each node periodically broadcasts its MANC packet; (**b**) Step 1: All one-hop neighbors successfully receive MANC packets, and node *d*, which occupies the last time slot #3, will consider a move to a new time slot. As node *d* can move to #3, it will include #3 in the switched time slot field of its MANC packet and broadcasts it in its reserved time slot, #5; (**c**) Node *d* checks the switched time slot fields of all MANC packets sent by its one-hop neighbors; (**d**) If all one-hop neighbors updated the information from node *d*, node *d* successfully acquires the new time slot, #3, reducing the length of the TDMA period in the next frame.

For instance, we consider one sample scenario shown in Figure 3. Nodes a,b,c, and *d* occupy time slots {2,1,3,4}, respectively. Figure 3a shows that each node periodically broadcasts its MANC packet during its occupied time slot. After all nodes receive the MANC packets, node *d*, which occupies the last time slot, considers a move to a new time slot to reduce the length of the TDMA period. Thus, node *d* can move to time slot #3, and node *d* includes #3 in the switched time slot field of its MANC packet and broadcasts in its reserved time slot #5, as shown in Figure 3b. Each node will broadcast its MANC packet including the information of the switched time slot field, #3, as shown in Figure 3c. If node *d* checks its switched time slot information in the MANC packets and all one-hop neighbors were updated, it successfully acquires the new time slot, reducing the length of the TDMA period in the next frame in Figure 3c.

### 3.2. Hybrid Time Slot Acquisition Scheme

In this section, we present a scheme that is used to occupy a time slot for a new node. After one duration of the TDMA period, a new node, *x*, receives all packets transmitted with its one-hop neighbors’ information. From the IDLE (IDLE is defined as the channel is detected as free) time slots, node *x* will consider occupying a time slot in the TDMA period. There are two cases: (1) there is at least one available time slot; and (2) there are no available time slots. Depending on these cases, we present two corresponding options.

When new node *x* enters the network, node *x* has to listen for one duration to collect and store information from one-hop neighbors. The parameters are stored to determine if there is an available time slot to access. Here, we propose a hybrid time slot–acquisition scheme in Algorithm 1. Let $T_{x}$ be a set of available time slots from the nodes. Node *x* checks $T_{x}$ to determine the status of available time slots. If $T_{x}$ is ∅, node *x* will broadcast a HELLO packet in the CSMA period. Node *x* checks the information in the packets transmitted by neighbors in MANC, to find out if it can successfully occupy a time slot or not. $N_{x}$, which is the one-hop neighbor set for node *x*, is collected when node *x* enters the network. 

**Algorithm 1:** Hybrid time slot acquisition scheme

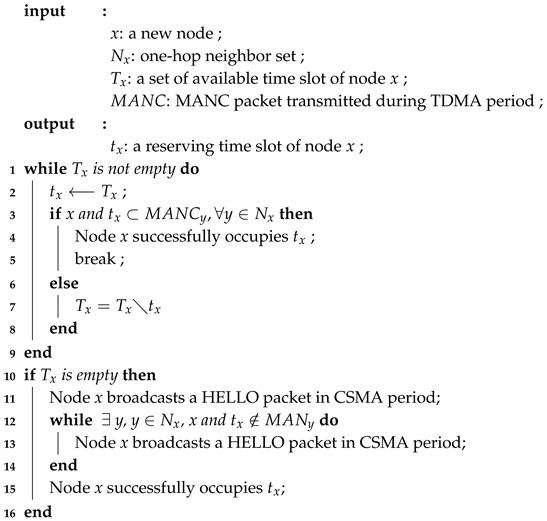



The main operations of the hybrid time slot acquisition scheme in the algorithm are as follows:

1. After node *x* receives all packets from its one-hop neighbor set, node *x* will find out if a time slot is available.
2. If there are available time slots, node *x* will randomly choose a time slot to occupy. In the next TDMA period, node *x* will broadcast its MANC packet in its reserved time slot. If all one-hop neighbors add node *x*’s ID and change the corresponding bit in the bitmap to 1 in their neighbor information of the MANC packets, node *x* successfully occupies the time slot. Otherwise, node *x* will choose another available time slot to occupy. If there are no remaining available time slots, node *x* will choose the next option.
3. If there are no available time slots, node *x* will broadcast a HELLO packet in the CSMA period. If all one-hop neighbors add node *x*’s ID, and change the extending time slot to in their neighbor information of the MANC packets, node *x* successfully occupies an extended time slot. Otherwise, node *x* will broadcast a HELLO packet again until it successfully occupies a time slot.

## 4. Model Analysis

First, we compared MANC packet delay with the maximum delay requirement. Second, we optimized time slot utilization on both the SCHs and the CCH under a saturated traffic load condition.

### 4.1. MANC Packet Delay

As described in Section 2, the size of the MANC packet transmitted by a node, *x*, is approximated as follows. The main part of the MANC packet consists of announcing the IDs in the one-hop neighbor set, $N_{\mathrm{nei}}$. If the maximum number of nodes that can exist in a one-hop neighbors set is $N_{\mathrm{nei}}^{\max}$, we need at least $\left\lceil \log_{2}N_{\mathrm{nei}}^{\max}\right\rceil$ bits to represent a node ID, where $\left\lceil .\right\rceil$ denotes the ceiling function. Therefore, the total MANC packet size (in bits), *S*, is

(1) $$\begin{array}{c} \begin{array}{c} S_{\mathrm{MANC}}=\left\lceil \log_{2}ID\right\rceil +\left\lceil \log_{2}s\right\rceil +\left\lceil \log_{2}s_{j}\right\rceil +S_{\mathrm{safe}}\\ +N_{\mathrm{nei}}\left(x\right).\left\lceil \log_{2}ID\right\rceil +\left\lceil \log_{2}s_{\mathrm{TDMA}}\right\rceil +S_{\mathrm{extra}}, \end{array} \end{array}$$

where ID is the ID of a node, *s* is the time slot used by node *x*, $s_{j}$ is the switched time slot if node *x* wants to switch to reduce the length of the TDMA period (denoted by $T_{\mathrm{TDMA}}$), and $s_{\mathrm{TDMA}}$ is the number of time slots. $S_{\mathrm{safe}}$ is the number of bits for a safety application packet. $S_{\mathrm{extra}}$ is the number of bits for all information in the packet, such as position, speed, and direction. We assume that *s*, $s_{j}$, and $s_{\mathrm{TDMA}}$ have the same number of bits, and the total number in one-hop set *N* is $N_{\mathrm{nei}}\left(x\right)+1$. Then, we can reduce Equation (1) to

(2) $$\begin{array}{c} \begin{array}{c} S_{\mathrm{MANC}}=\left\lceil \log_{2}ID\right\rceil N+3\cdot \left\lceil \log_{2}s\right\rceil +S_{\mathrm{safe}}+S_{\mathrm{extra}}. \end{array} \end{array}$$

### 4.2. The Efficient Multi-Channel MAC Protocol in VANETs

#### 4.2.1. Average Time to Successfully Make Reservation on the CCH

**Figure 4:**
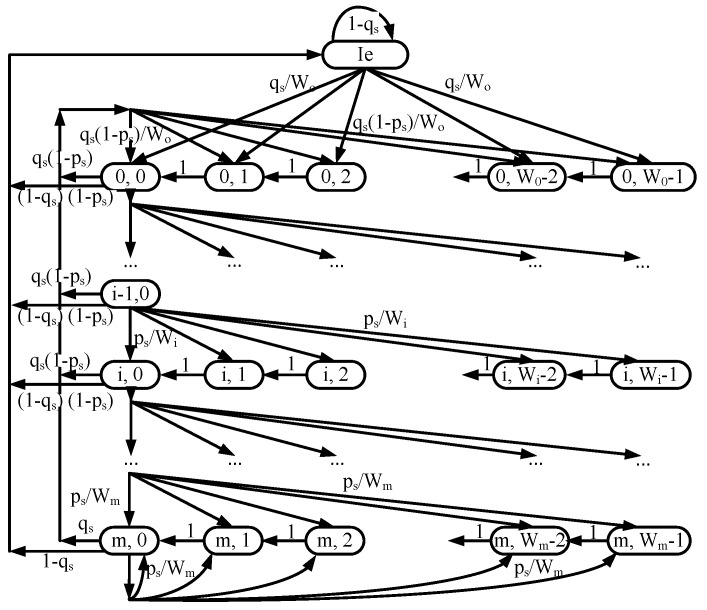
Markov chain of the WSA transmission.

The Markov model is known as an efficient tool to analyze the IEEE 802.11 distributed coordination function (DCF) method. In addition, the Markov chain used to analyze IEEE 802.11 DCF has been adopted under the IEEE 802.11p standard [20]. Under different data traffic densities, the Markov model can predict throughput and delay with high accuracy, in comparison with simulation results [13]. Now, we analyze the transmission of WSA packets by using the Markov chain. Let $b_{s}$*(t)* and $s_{s}$*(t)* be the stochastic processes representing the backoff window size and backoff state, respectively, for a given node in slot time *t*. Following [21], let *m* be the maximum backoff state, such that $W_{\max}$ = $2^{m}W_{0}$. $W_{i}$ is the maximal contention window (CW) of the ith backoff state, where, i∈(0,m), and $W_{i}$ = $2^{i}W_{0}$ ($W_{0}$ is the minimum contention window size). Let $p_{s}$ the probability of collision, where more than one node transmits in a single slot; let $I_{e}$ be the idle state with an empty buffer, and let $q_{s}$ be the probability of at least one new WSA packet in the buffer. Then, the bidimensional process {$s_{s}$*(t)*, $b_{s}$*(t)*} can be modeled with a discrete-time Markov chain, as shown in Figure 4. We assume that the generated packet arrives at the MAC layer in a Poisson manner with rate. Because there are two queues with the same arrival rate (CCHI and SCHI queues), the packet arrival rate of WSA packets at each node is $2\lambda_{s}$.

From the Markov chain and [22], the probability that a node transmits a WSA packet in an arbitrary time slot can be expressed as

(3) $$\begin{array}{c} \begin{array}{c} \tau_{s}=\frac{2\left(1-2p_{s}\right)q_{s}}{q_{s}\left[\left(W_{0}+1\right)\left(1-2p_{s}\right)+W_{0}p_{s}\left(1-{\left(2p_{s}\right)}^{m}\right)\right]+2\left(1-q_{s}\right)\left(1-p_{s}\right)\left(1-2p_{s}\right)}. \end{array} \end{array}$$

The collision probability, $p_{s}$, when more than one node transmits in the same time slot, is given by

(4) $$\begin{array}{c} \begin{array}{c} p_{s}=1-{\left(1-\tau_{s}\right)}^{N-1}. \end{array} \end{array}$$

Consequently, based on Equations (3) and (4), variables $\tau_{s}$ and $p_{s}$ can be solved by numerical methods. Note that $0\leq \tau_{s}\leq 1$ and $0\leq p_{s}\leq 1$.

In each time slot, let $P_{\mathrm{suc}}$ denote the probability of successful transmission of a WSA packet. A collision occurs on a channel with probability $P_{\mathrm{col}}$. We have

$$\left\{\begin{array}{c} P_{\mathrm{suc}}=N\cdot \tau_{s}\cdot {\left(1-\tau_{s}\right)}^{N-1},\\ P_{\mathrm{col}}=1-{\left(1-\tau_{s}\right)}^{N}-N\cdot \tau_{s}\cdot {\left(1-\tau_{s}\right)}^{N-1}. \end{array}\right.$$

**Figure 5:**
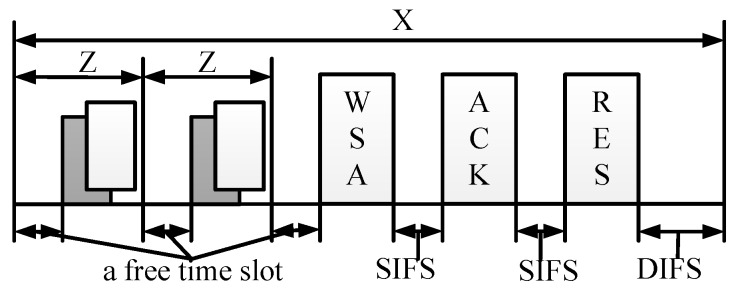
Contention model of making a reservation on the CCH.

Let $T_{\mathrm{WSA}}$, $T_{\mathrm{RES}}$, and $T_{\mathrm{ACK}}$ denote the time for transmitting a WSA, RES, and an ACK, respectively. $T_{\mathrm{SIFS}}$, $T_{\mathrm{DIFS}}$, and δ are the short inter-frame space (SIFS) time, distributed coordination function inter-frame space (DIFS) time, and propagation time, respectively. Hence, the duration of a free time slot, a transmission collision, and a successful reservation are $T_{\mathrm{idle}}$, $T_{\mathrm{col}}$, and $T_{\mathrm{suc}}$, respectively. Then, from Figure 5, we have

$$\left\{\begin{array}{c} T_{\mathrm{idle}}=aSlotTime,\\ T_{\mathrm{col}}=T_{\mathrm{WSA}}+\delta +T_{\mathrm{DIFS}},\\ T_{\mathrm{suc}}=T_{\mathrm{WSA}}+T_{\mathrm{RES}}+2\cdot T_{\mathrm{SIFS}}+T_{\mathrm{ACK}}+3\cdot \delta +T_{\mathrm{DIFS}}. \end{array}\right.$$

According to [21], let *X* represent the time interval from control channel access contention to the time a reservation is successfully made. *Z* denotes the interval between two free time slots before a reservation is successfully made. All *X* and *Z* are depicted in Figure 5. From [21], the mean of time interval *X* is given by

(5) $$\begin{array}{c} E\left[X\right]=T_{\mathrm{idle}}/P_{\mathrm{suc}}+P_{\mathrm{col}}\cdot T_{\mathrm{col}}/\;P_{\mathrm{suc}}+T_{\mathrm{suc}}. \end{array}$$

From the mean of time interval *X*, the probability of $q_{s}$ can be approximated as

(6) $$\begin{array}{c} q_{s}=1-e^{-2\lambda_{s}\cdot E\left[X\right]}. \end{array}$$

If $\lambda_{s}$ goes to infinity, this means that all nodes providing service always have available WSA packets, which is saturated throughput [21].

#### 4.2.2. Optimization of Time Slot Selection in the TDMA Period and on SCHs under the Saturated Traffic Load Condition

According to [21] and the definition of saturated traffic load, let $G_{1}$ be the number of reservations made on the control channel during the CSMA period, and let $G_{2}$ be the number of time slots, $N_{\mathrm{SCH}}$, on all SCHs during the CCHI and SCHI. The length of the CSMA period, $T_{\mathrm{CON}}$, is given as

$$\begin{array}{c} T_{\mathrm{CON}}=\frac{N_{\mathrm{sch}}\cdot \left(\frac{1}{P_{\mathrm{suc}}}T_{\mathrm{idle}}+\frac{P_{\mathrm{col}}}{P_{\mathrm{suc}}}T_{\mathrm{col}}+T_{\mathrm{suc}}\right)\cdot \left(T_{\mathrm{total}}-T_{\mathrm{TDMA}}\right)}{N_{\mathrm{sch}}\cdot \left(\frac{1}{P_{\mathrm{suc}}}T_{\mathrm{idle}}+\frac{P_{\mathrm{col}}}{P_{\mathrm{suc}}}T_{\mathrm{col}}+T_{\mathrm{suc}}\right)+s_{\mathrm{SCH}}}, \end{array}$$

where $T_{\mathrm{total}}$ is a sync interval of 50 ms, and $s_{\mathrm{SCH}}$ is the length of one time slot on the SCHs. However, we can obtain $G_{1}$ from $T_{\mathrm{CON}}$ as follows:

(7) $$\begin{array}{c} G_{1}=\frac{T_{\mathrm{CON}}}{E[X]}. \end{array}$$

In Figure 1, the number of time slots in both the TDMA period and all the SCHs will be optimized to comply with the saturated traffic load condition. If $G_{1}$ is known, we can calculate the optimal value of $G_{2}$, or vice versa. This will be discussed in the next section.

#### 4.2.3. Optimization of $G_{1}$ Based on a Known $G_{2}$

From Equations (5) and (7), we observe $T_{\mathrm{CON}}$, and hence, $T_{\mathrm{TDMA}}$ is calculated as $T_{\mathrm{total}}-T_{\mathrm{CON}}$. Based on Equation (2), we can calculate the number of time slots in the TDMA period, $s_{\mathrm{TDMA}}$, to satisfy maximum delay requirement. This result is obtained by solving the following equation:

(8) $$\begin{array}{c} \begin{array}{c} \frac{T_{\mathrm{CON}}}{s_{\mathrm{TDMA}}}=\left\lceil \log_{2}ID\right\rceil N+3\cdot \left\lceil \log_{2}s_{\mathrm{TDMA}}\right\rceil +S_{\mathrm{safe}}+S_{\mathrm{extra}}. \end{array} \end{array}$$

#### 4.2.4. Optimization of $G_{2}$ Based on a Known $G_{1}$

We assume that $N=N_{\mathrm{nei}}^{\max}$ and $S_{\mathrm{MANC}}$ are known. We can obtain the length of the TDMA period, $T_{\mathrm{TDMA}}=N\cdot S_{\mathrm{MANC}}$. Hence, the length of the CSMA period is $T_{\mathrm{CON}}=T_{\mathrm{total}}-T_{\mathrm{TDMA}}$. From Equation (7), we obtain $G_{1}$. In the saturated traffic load condition, we can optimize the number of time slots in all SCHs in which the condition is $G_{2}=G_{1}$. This result is given as

(9) $$\begin{array}{c} G_{2}=G_{1}=\frac{T_{\mathrm{CON}}}{E[X]}. \end{array}$$

#### 4.2.5. Saturated Throughput

According to [21], the saturated throughput is given by

(10) $$\begin{array}{c} S_{SCH}=\frac{T_{\mathrm{SCH}}}{E[T_{data}]}\cdot N_{SCH}\cdot V, \end{array}$$

where $N_{SCH}$ represents the number of available SCHs in a VANET, and V is the payload of the service packet.

We assume that the length of the service packet is constant. Hence, the duration for transmitting a service packet on an SCH is given by

(11) $$\begin{array}{c} T_{data}=T_{h}+T_{e}+T_{SIFS}+T_{ACK}+T_{DIFS}, \end{array}$$

where $T_{h}$ is the cost of the MAC and physical layer headers introduced by the service data packet, $T_{e}=V/R_{SCH}$, and $R_{SCH}$ is the data rate of the CCH.

## 5. Model Validation

To validate our model, we use an event-driven simulation written in Matlab (R2017b, MathWorks). The values of the parameters to obtain the numerical result from the analytical model are in Table 3. In our model, we fix the WSA packet arrival rate, $\lambda_{s}$, at 25 packets per second. We also assume that in the CSMA period there are $N^{\max}=100$ neighbor nodes, which always have available WSA packets. In our model, time slot allocation operates similarly to HTC-MAC [17]. Each node had successfully acquired a time slot in the TDMA period. Based on Section 4, we present two performance evaluations here: the efficient multi-channel MAC protocol and time slot utilization.

### 5.1. Performance of Efficient Multi-Channel MAC Protocol

Here, we define two key performance indicators to evaluate the different protocols.

1. Protocol overhead, and packet delay.
2. Time slot acquisition rate (the number of nodes that successfully occupy time slots to the total number of nodes).
3. Packet delivery ratio of the safety packets (the number of successful safety packet transmissions to all transmitted WSA packets). Safety packet transmission is considered successful if an RSU successfully receives the safety packets sent.
4. Packet delivery ratio of the WSA packets (the number of successful WSA packet transmissions to all transmitted WSA packets). WSA packet transmission is considered successful if a sender successfully receives an ACK for the packet sent.

#### 5.1.1. Protocol Overhead and Packet Delay

As done in [4], we make the following assumptions: $N^{\max}$ = 100, data rate *R* = 12 Mbps supported by the IEEE 802.11p orthogonal frequency-division multiplexing (OFDM) physical layer for the 5 GHz band, ID = 1 byte, *s* = 100 time slots, $S_{\mathrm{safe}}$ = 200 bytes, and $S_{\mathrm{extra}}$ = 30 bytes. Furthermore, we can see the MANC packet sizes, $S_{\mathrm{MANC}}$, in Table 4. After adding the guard period and physical layer header, we assume a duration of $T_{\mathrm{slot}}$ ms. Consequently, with *s* = 100 time slots, the duration of one complete frame on the control channel is $T_{\mathrm{comp}}$, as shown in Table 4. The maximum allocated latency is 100 ms [5]. In Table 4, a node can transmit its safety application packets once every $T_{\mathrm{comp}}$, which complies with the maximum delay requirements. The duration of one complete frame on the control channel using MANC packets is less than an ANC packet under HTC-MAC.

#### 5.1.2. Time Slot Acquisition Rate

**Figure 6:**
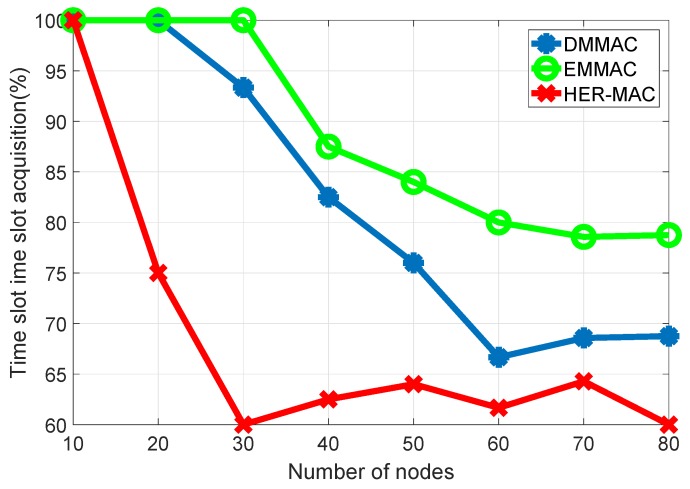
Time slot acquisition rate.

In a TDMA-based period, each vehicle must acquire at least one time slot. Our protocol allows a new vehicle to occupy an available time slot in a flexible way, according to Section 3.2. EMMAC can reduce the access collisions that occur in TDMA-based access schemes. Access collisions are defined as collisions that occur among nodes that are trying to occupy the same time slot [4]. On the other hand, DMMAC designs virtual time slots for new nodes to access. However, the number of virtual time slots is limited and few in number. Hence, access collisions occur under DMMAC. Otherwise, in the HER-MAC protocol, a new time slot must broadcast a HELLO packet in the contention period to access a time slot. The probability of HELLO packet collision is higher than under the EMMAC and DMMAC protocols. The reason is that there are many types of packets transmitted in the contention period under the HER-MAC protocol, such as SWITCH, WSA, and ACK packets. Consequently, the time slot acquisition rate in the EMMAC protocol is higher than both HER-MAC and DMMAC protocols, as shown in Figure 6.

#### 5.1.3. Packet Delivery Ratio of Safety Packets

**Figure 7:**
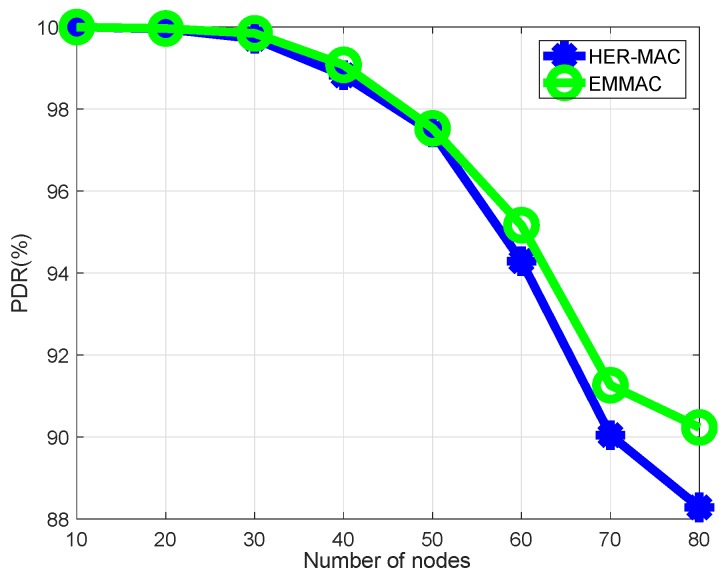
Packet delivery ratio of safety packets.

Both EMMAC and DMMAC protocols allow each vehicle that has a safety packet to transmit the safety packet during its occupied time slot. When the number of nodes increases, the merging collisions also increase because of the moving nodes. Merging collisions are defined as collisions that occur among nodes that have successfully acquired a time slot. In VANETs, merging collisions can happen due to acceleration or deceleration among vehicles moving in the same direction [4]. Nevertheless, HER-MAC allows each vehicle that has a safety packet to transmit that safety packet during the contention period. Thus, in the contention period under the HER-MAC protocol, there are many types of packets transmitted, such as SWITCH, WSA, and ACK packets, and the packet delivery ratio (PDR) for safety packets is lower than in our proposal, as shown in Figure 7.

#### 5.1.4. Packet Delivery Ratio of WSA Packets

**Figure 8:**
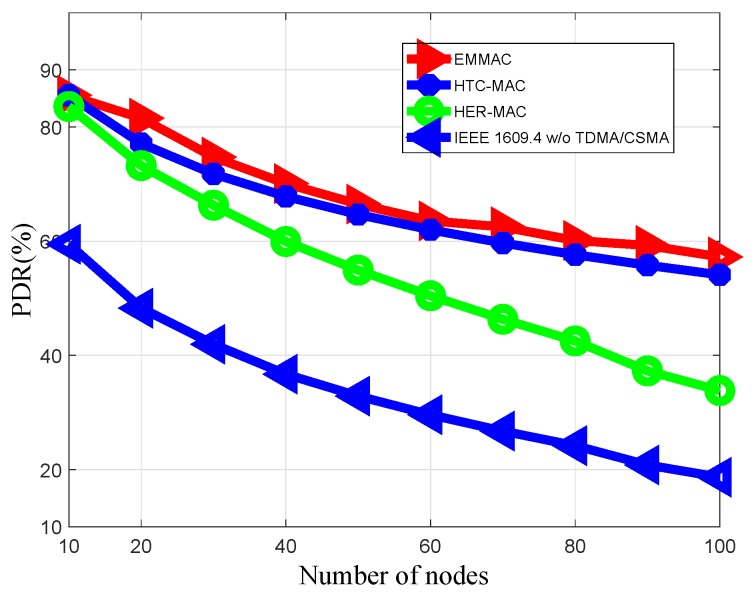
Packet delivery ratio of WSA packets.

Under HER-MAC, there are three types of packets transmitted: HELLO, emergency, and WSA packets. The emergency packet has the highest priority, while HELLO and WSA packets have a lower priority. Because all HELLO, emergency, and WSA packets are transmitted in the CSMA period with different priorities, the transmission probability for WSA packets will decrease when the number of HELLO and emergency packets increases [23]. Consequently, the PDR of WSA packets also is better than under HTC-MAC, HER-MAC, and IEEE 1609.4, as shown in Figure 8.

### 5.2. Time Slot Utilization

#### 5.2.1. Optimization of Time Slot Selection

We consider the first case in which $G_{2}$ is known. Based on Equation (8), we obtain the number of neighbors and the number of time slots in the TDMA period. The smaller $G_{2}$, the greater $s_{\mathrm{TDMA}}$ are shown in Table 5. As $G_{2}$ increases, the number of time slots in the TDMA period, $s_{\mathrm{TDMA}}$, will decrease. Furthermore, if $G_{2}$ is fixed, the number of time slots in the TDMA period has little effect on variable *N*.

In the second case, we fix the number of neighbors. By using Equations (2) and (9), we can obtain the number of time slots in the TDMA period and the number of time slots in all SCHs. As the number of neighbors increases, the number of time slots in all SCHs decreases, as shown in Table 6. When the length of the TDMA period is greater by increasing the MANC packet size, the length of the CSMA period will decrease. Finally, the number of reservations successfully made on the CCH during the CSMA period decreases, and the number of time slots also decreases in all SCHs.

**Figure 9:**
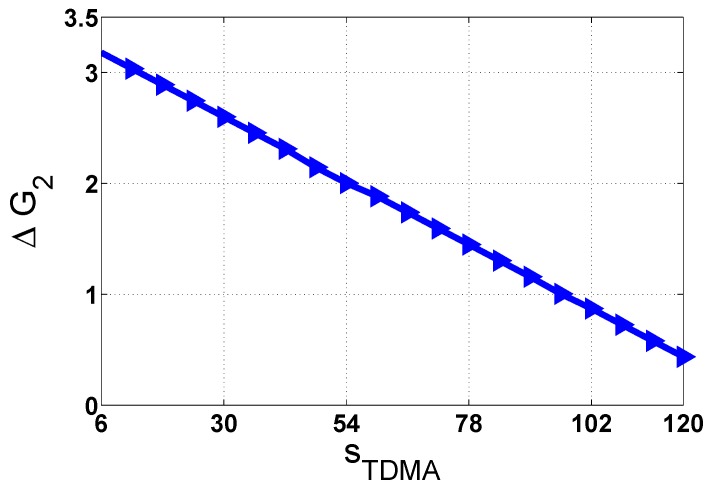
The difference of $G_{2}$ at $N^{\max}=100$ and $N^{\min}=10$.

Now, we trade off between three values: the number of the time slots in all SCHs ($G_{2}$), the number of neighbors (*N*) and the number of time slots in the TDMA period ($s_{\mathrm{TDMA}}$), as shown in Figure 9. In Table 5 and Figure 9, with $N^{\max}=100$, and $N^{\min}=10$, when $s_{\mathrm{TDMA}}$ increases, the difference of the number of time slots in SCHs (defined by $\Delta G_{2}$) very low. Hence, we can initialize the number of time slots with $N^{\min}$ under the saturated traffic load condition, such as $N^{\min}=10$ in Table 6. Then, based on node density, the length of RP can be changed by broadcasting the MANC packet.

#### 5.2.2. Saturated Throughput

Now, we compare the throughput of time slot selection under different levels of time slot utilization. When $s_{\mathrm{TDMA}}$ increases, the difference in the number of time slots in the SCHs (defined by $\Delta G_{2}$) is very low, as shown in Figure 9. The number of nodes, *N*, varies from 40 to 80, and the *N* nodes providing service always have available WSA packets. We modified the time slot utilization and compared that with the analytical results in Equation (10).

**Figure 10:**
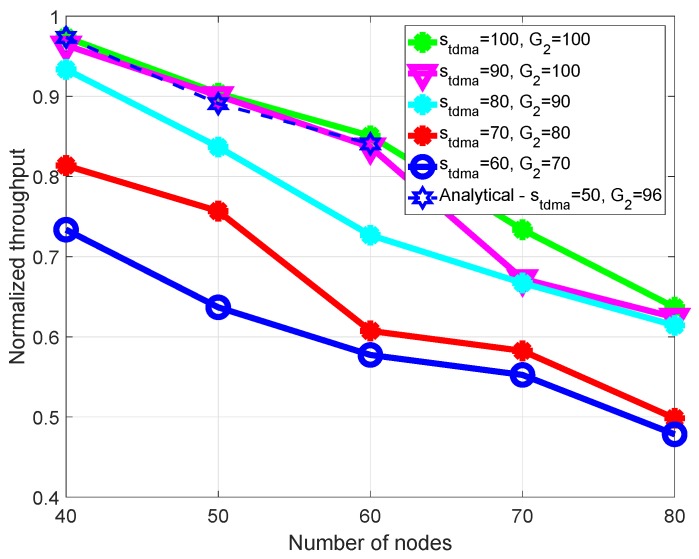
Normalized throughput.

Figure 10 shows the saturated throughput in terms of the number of nodes and different levels of time slot utilization. Clearly, the saturated throughput is affected by time slot utilization. If the time slot acquisition rate is fast, then the number of TDMA-based periods is reduced, so the saturated throughput increases. Consider node 40 in Figure 10; the saturated throughput of the EMMAC protocol using $s_{\mathrm{TDMA}}=\left\{100,90,80\right\}$ is greater than from using $s_{\mathrm{TDMA}}=\left\{70,60\right\}$. This is because, when all nodes occupied time slots, they reduced the length of the TDMA-based period, as explained in Section 3.1. Consequently, the SCHI is increased, and it can offer the chance for more contention-free transmissions of service packets. When $s_{\mathrm{TDMA}}$ is greater than, or equal to, the number of nodes, the normalized throughput is higher. For instance, when the number of nodes is 80, the normalized throughput using $s_{\mathrm{TDMA}}=\left\{100,90,80\right\}$ is greater than using $s_{\mathrm{TDMA}}=\left\{70,60\right\}$, as shown in Figure 10. When the number of nodes changes from 40 to 60, and the EMMAC protocol uses $s_{\mathrm{TDMA}}=100,90$, the number of one-hop neighbors has little effect on saturated throughput. Our analytical result is close to the simulation result.

## 6. Conclusions

This paper proposed a multi-channel MAC protocol with a modified announcement packet transmitted in the TDMA period to reduce transmission overhead. The results show that the delay and packet delivery ratio are slightly better than under HER-MAC and IEEE 1609.4. Simulation results show that the proposed algorithm can achieve up to 26% and 38% performance gains in terms of packet delivery ratio of WSA packets, in comparison with HER-MAC and IEEE 1609.4, respectively. We use a Markov chain and a stochastic process to establish the number of time slots in both the TDMA period and in the SCHs under a condition of saturated traffic load, which has little effect on the number of neighbors. However, the probability of all nodes acquiring time slots decreases when the number of time slots is less than the number of neighbors.

## Figures and Tables

**Table 1:** DSRC data traffic requirements [10,11].

Priority	Network Traffic Type	Application	Allowable Latency (ms)	Packet Size (Bytes)/Bandwidth
Safety of life	Event	Intersection collision warning/avoidance	∼100	∼100
Safety of life	Event	Emergency vehicle warning	∼100	∼100/∼10 Kbps
Safety of life	Periodic	Cooperative collision warning	∼100	∼100/∼10 Kbps
Safety of life	Periodic	Speed limits notification	∼100	∼100/∼10 Kbps
Safety of life	Periodic	Traffic ligh speed advisory/violation	∼100	∼100/∼10 Kbps
Safety	Event	Transit vehicle signal priority	∼1000	∼100
Safety	Periodic	Work zone warning	∼1000	∼100/∼1 Kbps
Non-safety	Event	Toll collection	∼50	∼100
Non-safety	Periodic	Service announcements	∼500	∼100/∼2 Kbps
Non-safety	-	Movie download (2 h of MPEG 1): 10 min download time	N/A	∼100/>20 Kbps

**Table 2:** Comparison of hybrid MAC protocols in VANET.

Name	Published	TDMA Period Adjustment	Optimized Interval	Advantages	Disadvantages
DMMAC [15]	2010	Yes	No	- The safety packets under various traffic conditions guarantee transmission delay - Provides collision-free transmission.	- Simulations of DMMAC are carried out on straight road scenarios with a smaller number of time slots than the number of vehicles - Access and merging collisions degrade the performance of DMMAC under various traffic conditions.
HER-MAC [16]	2014	Yes	No	- Improves non-safety packet delivery ratio and throughput.	- The throughput on the CCH decreases due to the control overhead. - The operation needs a high level of coordination.
HTC-MAC [17]	2016	Yes	No	- HTC-MAC eliminates HELLO packets. - HTC-MAC outperforms HER-MAC in terms of the average number of nodes that acquire a time slot.	- HTC-MAC also requires a large overhead due to the periodic broadcasting of ANC messages.
EFAB [18]	2017	Yes	No	- Improves broadcast safety packets. - Higher safety packet delivery ratio on the CCH.	- Does not consider a scenario where the control vehicle leaves.
CS-TDMA [19]	2014	Yes	No	- Reduces transmission delay and packet collision rate.	- The use of a GPS and a digital map makes this system expensive.
Our proposal	-	Yes	Yes	- Improves system throughput for non-safety packets. - Trade-off between time slot selection in both the TDMA period and on SCHs under a saturated traffic load condition.	- They require a pre-determined channel access.

**Table 3:** Parameter settings.

Parameters	Value	Parameters	Value
Data rate of each channel	3 Mbps	Number of SCH	4
Highway length	1 km	Lane width	5
Lanes	4	Direction	2
Speed mean	100 km/h	Speed deviation	20 km/h
#slot for TDMA period	10 to 100	Transmission range	150 m
Data rate	12 Mbps	ACK	14 bytes
WSA	100 bytes	RES	14 bytes
Slot time σ	13 μs	SIFS	32 μs
Propagation time δ	1 μs	DIFS	58 μs
$\lambda_{s}$	25 pkts/s	$W_{0}$	16
MAC header	256 bits	$W_{s}$	64
Service packet length	256 bits	PHY header	192 bits

**Table 4:** Manc packet delay in Emmac protocol.

N(x)	$S_{\mathrm{MANC}}$	$T_{\mathrm{trans}}$	$T_{\mathrm{comp}}$	$T_{\mathrm{comp}}^{\mathrm{HTC-MAC}}$
10	1891	0.16	20.76	21.28
20	1921	0.16	21.01	22.12
40	1981	0.17	21.51	23.75
60	2041	0.17	22.01	25.45
80	2101	0.18	22.51	27.12
100	2161	0.18	23.01	28.78

**Table 5:** VALUE $s_{\mathrm{TDMA}}$.

	N	40	50	60	70	80	90	100
$G_{2}$								
6		24	23	23	23	22	22	22
30		19	19	19	18	18	18	18
60		14	14	13	13	13	13	13
90		8	8	8	8	8	8	8
120		3	3	3	3	3	3	3

**Table 6:** VALUE $G_{2}$.

	$s_{\mathrm{TDMA}}$	40	50	60	70	80	90	100
*N*								
10		114	108	102	96	90	84	78
20		114	108	102	96	90	84	78
40		114	108	102	96	90	84	78
60		x	x	96	90	84	78	72
80		x	x	x	x	x	78	72

## Abbreviations

The following abbreviations are used in this manuscript:

VANET	Vehicular ad-hoc network
QoS	Qualiy of service
TDMA	Time-division multiple access
CSMA	Carrier-sense multiple access
CCH	Control channel
SCH	Service channel
DSRC	Dedicated short range communication
V2V	Vehicle-to-vehicle communication
V2R	Vehicle-to-RSU communication
WAVE	Wireless access in vehicular environments
MAC	Medium access control
GPS	Global positioning system
WSA	WAVE service announcement
MANC	The modified announcement packet
CCHI	Control channel Interval
SCHI	Service channel interval
UTC	Coordinated universal time
MAL	Maximum active length of vehicles
ABFL	Adaptive broadcast frame length
OL	Maximum ABFL within a one-hop area
EMMAC	Efficient multi-channel MAC protocol in VANETs
TP	TDMA-based period
CP	Contention period
SIFS	Short inter-frame space
DCF	Distributed coordination function
DIFS	DCF inter-frame space
